# Supplementary material for: Reversing food preference through multisensory exposure
Source: PLoS One. 2023 Jul 20;18(7):e0288695. doi: 10.1371/journal.pone.0288695 (PMC10359010; doi:10.1371/journal.pone.0288695)
Supplement: S1 File — (PDF) [file pone.0288695.s001.pdf]

Supporting information

S1 Appendix LFE, NEC and DFE class formation: At first, we have calculated the difference (Δ_i) between the mean value ratings of chocolate and chips for all subjects i. Then we put i th subject in NEC if Δ_i lies between the interval $(-\frac{s}{\sqrt{n}}t_{(\text{n-1}),\frac{\alpha}{2}}, \frac{s}{\sqrt{n}}t_{(\text{n-1}),\frac{\alpha}{2}})$ else in the DFE or LFE class, where $n = 96$ (number of participants), s = standard deviation of Δ (which was found to be 2.72) and $t_{(\text{n-1}),\frac{\alpha}{2}}$ is the $(1-\frac{\alpha}{2})$th quantile of t-distribution with $(n-1)$ degrees of freedom. In our analysis we have chosen $\alpha = 0.05$.

	Shorter duration $N = 18$	Longer duration $N = 17$	Test Statistic
YES craving	72% (13)	65% (11)	$\chi^2(1) = 0.2292,\ P = 0.6321$
NO craving	28% (5)	35% (6)	

Table S1. Time duration difference in response to question of whether food cravings had ever been experienced. This analysis is done based on the behavioral data of the participants who took part in EEG experiment.

S2 Appendix: Time duration of refrained from eating: The chi-square (χ^2) test of independence was performed to test the dependency between time duration of refrained from eating and the inducement of craving on the subjects performing the EEG experiment ($N = 35$) based on a 2×2 contingency table (see Table S1). Two classes (namely, Shorter duration and Longer duration) were formed based on the median time duration (found to be 193 min). Based on the median value, two classes, namely, Shorter duration (120 min $\leq$ time $\leq$ 193 min) and Longer duration ($>$ 193 min) were formed. A participant was classified in the Shorter duration class (or Longer duration class) if the participant's time duration of refrained from eating belongs to (120, 193] (or (193, 360]).

The test confirmed that there is no reason to believe that food craving is dependent on the time duration of refrained from eating ($\chi^2(1) = 0.2292$, p-value = 0.6321, see Table S1).

S3 Appendix. Spline Regression Model (SRM): A kth degree spline regression model with h knots, $t_1 < t_2 < ... < t_h$, with no continuity restriction is given by:

$$y = \alpha + \sum_{j=1}^{h+1}\sum_{i=0}^{k} \beta_{ij} C_j(x) x^i + \epsilon, \tag{5}$$

where α and β_{ij} are the coefficients of the model and ϵ follows $N(0, \sigma^2)$. $C_j(x)$ is defined as follows:

$$\begin{aligned} C_0(x) &= I(x < t_1) \\ C_1(x) &= I(t_1 \leq x < t_2) \\ &\vdots \\ C_{h-1}(x) &= I(t_{h-1} \leq x < t_h) \\ C_{h-1}(x) &= I(t_h \leq x) \end{aligned}$$

S4 Appendix. Bonus amount calculation Becker-Degrot-Marschak (BDM) is a commonly used auction procedure to elicit willingness-to-pay. In general, under the BDM, a participant announces a bid for an item; the item's price is then randomly drawn. If the bid amount exceeds the price, the participant gets the item and pays the drawn price. If the bid amount is less than the drawn price, the participant does not receive the item and pays nothing.

In each trial of our study, the proportion of the bid amount out of the left amount was calculated. Then a random number between 0 and 1 was generated. If this proportion exceeded the random number, the participants won an extra unit of ₹10 and if the proportion was less than the random number, the participant got no extra unit. Then a random block was selected for each participant and total number of the won unit was counted for that block. A subject received ₹100 + number of won unit× ₹10.

Bonus amount was calculated in the similar fashion for the EEG studies. But each of the subjects for the EEG study was compensated at the consolidated rate of Rs. ₹400 + number of won unit× ₹10.

S1 Fig: Scatter plots of DFE and LFE:

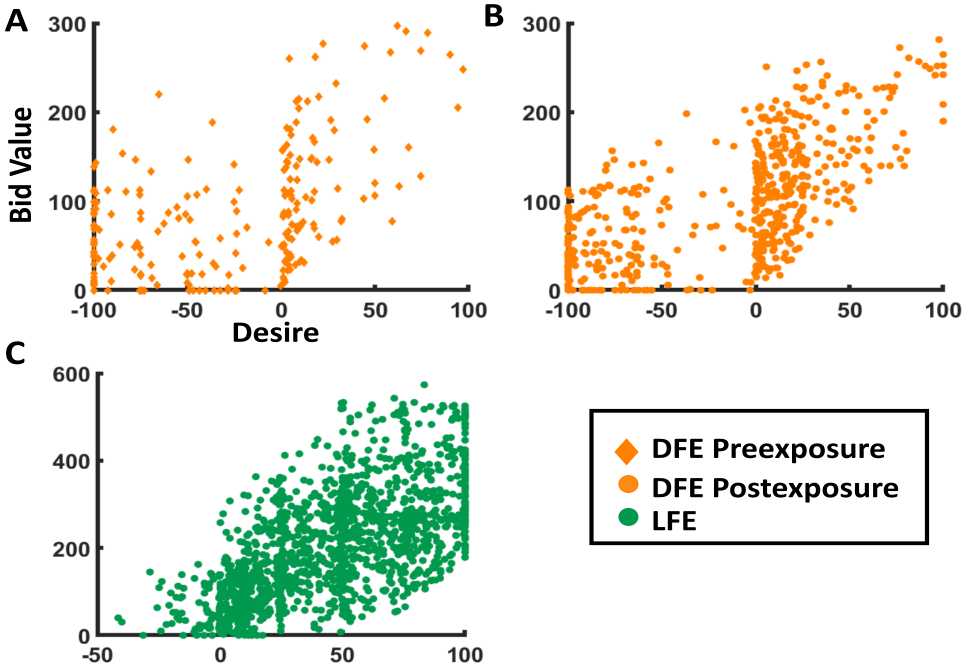

Fig S1. Scatter plots of DFE and LFE. In order to visualise the data well, bid value proportion is multiplied by 1000 A: DFE at Preexposure B: DFE at Postexposure C: LFE

S3 Appendix: Models for Behavioral Data:

DFE Models:

Model 1:

$$BV_{\text{Pre}} = (\alpha_0 + \alpha_1 DV_{\text{Pre}}) I(DV_{\text{Pre}} < 0) + (\alpha_2 + \alpha_3 DV_{\text{Pre}}) I(DV_{\text{Pre}} \geq 0) + \alpha_4 RT_{\text{Pre}} + \epsilon \quad (6)$$

$$BV_{\text{Post}} = (\beta_0 + \beta_1 DV_{\text{Post}}) I(DV_{\text{Post}} < 0) + (\beta_2 + \beta_3 DV_{\text{Post}}) I(DV_{\text{Post}} \geq 0) + \beta_4 RT_{\text{Post}} + \epsilon \quad (7)$$

Model 2:

$$\begin{aligned} BV_{\text{Pre}} =& (\alpha_0 + \alpha_1 DV_{\text{Pre}}) I(DV_{\text{Pre}} < 0) + (\alpha_2 + \alpha_3 DV_{\text{Pre}}) I(DV_{\text{Pre}} \geq 0) + \alpha_4 RT_{\text{Pre}} \\ &+ \alpha_5 I(E_{\text{type}} = Sweet) + \epsilon \end{aligned} \quad (8)$$

$$\begin{aligned} BV_{\text{Post}} =& (\beta_0 + \beta_1 DV_{\text{Post}}) I(DV_{\text{Post}} < 0) + (\beta_2 + \beta_3 DV_{\text{Post}}) I(DV_{\text{Post}} \geq 0) + \beta_4 RT_{\text{Post}} \\ &+ \beta_5 I(E_{\text{type}} = Sweet) + \epsilon \end{aligned} \quad (9)$$

Model 3:

$$\begin{aligned} BV_{\text{Pre}} =& (\alpha_0 + \alpha_1 DV_{\text{Pre}}) I(DV_{\text{Pre}} < 0) + (\alpha_2 + \alpha_3 DV_{\text{Pre}}) I(DV_{\text{Pre}} \geq 0) + \alpha_4 RT_{\text{Pre}} \\ &+ \alpha_5 I(E_{\text{type}} = Sweet) + \alpha_6 I(E_{\text{type}} = Sweet) \times DV + \epsilon \end{aligned} \quad (10)$$

$$\begin{aligned} BV_{\text{Post}} =& (\beta_0 + \beta_1 DV_{\text{Post}}) I(DV_{\text{Post}} < 0) + (\beta_2 + \beta_3 DV_{\text{Post}}) I(DV_{\text{Post}} \geq 0) + \beta_4 RT_{\text{Post}} \\ &+ \beta_5 I(E_{\text{type}} = Sweet) + \beta_6 I(E_{\text{type}} = Sweet) \times DV + \epsilon, \end{aligned} \quad (11)$$

where, BV_i, DV_i & RT_i denote the bid value, desire value and reaction time of bid value, respectively, at preexposure or at postexposure depending on $i = Pre$ or $Post$, $I(E_{type} = Sweet)$ is an indicator function, whose value is 1 if exposure type is sweet item and 0 if the exposure type is savory item and α's and β's are the coefficients of the DFE models 1,2, and 3 as described above.

LFE Models:

Model 1:

$$BV = (\gamma_0 + \gamma_1 DV) I(DV < 0) + (\gamma_2 + \gamma_3 DV) I(DV \geq 0) + \gamma_4 RT + \epsilon \quad (12)$$

Model 2:

$$\begin{aligned} BV =& (\gamma_0 + \gamma_1 DV) I(DV < 0) + (\gamma_2 + \gamma_3 DV) I(DV \geq 0) + \gamma_4 RT \\ &+ \gamma_5 I(E_{\text{type}} = Sweet) + \epsilon \end{aligned} \quad (13)$$

Model 3:

$$\begin{aligned} BV =& (\gamma_0 + \gamma_1 DV) I(DV < 0) + (\gamma_2 + \gamma_3 DV) I(DV \geq 0) + \gamma_4 RT \\ &+ \gamma_5 I(E_{\text{type}} = Sweet) + \gamma_6 I(E_{\text{type}} = Sweet) \times DV + \epsilon. \end{aligned} \quad (14)$$

In the above LFE models, BV, DV & RT denote the bid value, desire value and reaction time of bid value, respectively. $I(E_{type} = Sweet)$ is an indicator function, whose value is 1 if exposure type is sweet item and 0 if the exposure type is savory item and α's and β's are the coefficients of the LFE models 1,2, and 3 as described above.

NEC model:

Model 1:

$$BV = \delta_0 + (\delta_1 DV) I(t \leq 15)) + (\delta_2 DV) I(t > 15)) + \delta_3 RT + \epsilon \tag{15}$$

Model 2:

$$\begin{aligned} BV =& \delta_0 + (\delta_1 DV) I(t \leq 15)) + (\delta_2 DV) I(t > 15)) + \delta_3 RT \\ &+ \delta_4 I(E_{\text{type}} = Sweet) + \epsilon \end{aligned} \tag{16}$$

Model 3:

$$\begin{aligned} BV =& \delta_0 + (\delta_1 DV) I(t \leq 15)) + (\delta_2 DV) I(t > 15)) + \delta_3 RT \\ &+ \delta_4 I(E_{\text{type}} = Sweet) + \delta_5 I(E_{\text{type}} = Sweet) \times DV + \epsilon, \end{aligned} \tag{17}$$

where BV, DV, RT & t denote the bid value, desire value, reaction time of bid value and trial number respectively, for the NEC group and δ's are the coefficients of the model. As described in the DFE and LFE models, $I(E_{type} = Sweet)$ denotes an indicator function, whose value is 1 if exposure type is sweet item and 0 if the exposure type is savory item.

S2 Table: Model Selction Table:

Model Selection : DFE Preexposure				
Models	Adjusted R^2	AIC	BIC	Significant covariates
Model 1(6)	0.7486	2583.94	2604.75	Intercepts, Desire value in [0,100]
Model 2(8)	0.6408	2800.21	2824.55	Intercepts, Desire value in [0,100]
Model 3(10)	0.6389	2823.82	2851.70	Intercepts, Desire value in [0,100]
Model Selection : DFE Postexposure				
Model 1(7)	0.8319	5978.07	6004.09	Intercepts, Desire value in [-100,0) and in [0,100]
Model 2(9)	0.8315	5980.25	6010.61	Intercepts, Desire value in [-100,0) and in [0,100]
Model 3(11)	0.8314	5981.74	6016.44	Intercepts, Desire value in [-100,0) and in [0,100]
Model Selection : LFE				
Model 1(12)	0.8304	13591.63	13621.58	Intercepts, Desire value in [0,100]
Model 2(13)	0.7686	14830.18	14865.39	Intercept and Desire value in [0,100]
Model 3(14)	0.7676	15284.38	15324.84	Intercept and Desire value in [0,100]
Model Selection : NEC				
Model 1(15)	0.5946	14347.42	14372.83	Intercept, Desire in preexposure and postexposure, Reaction time
Model 2(16)	0.4121	18041.78	18073.15	Intercept, Desire in preexposure and postexposure, Reaction time, Exposed food type
Model 3(17)	0.4186	18027.32	18063.92	Intercept, Desire in preexposure and postexposure, Reaction time, Interaction between desire and exposed food type

Table S2. Model selection: Adjusted R- squared value, AIC, BIC and significant covariates of each of the models are depicted. Based on the AIC and BIC values we have selected Model 1 (highlighted in pink).

S3 Tables: Tables related to EEG data analysis: Tables S3, S4, S5 and S6 provided the localization effect of the ERP components P200, N200, P300 and LPP, respectively.

	**Preexposure**		**Postexposure**	
	**Mean difference, t-value**	$p-$**value**	**Mean difference, t-value**	$p-$**value**
**DFE**	$-1.8634, t_{11} = -1.6176$	0.0670	$-0.6241, t_{11} = -1.0130$	0.1664
**LFE**	$-1.0627, t_{11} = -0.9043$	0.1926	$0.0081, t_{11} = 0.0241$	0.4906
**NEC**	$-0.0071, t_{10} = -0.0116$	0.4955	$0.3313, t_{10} = 1.01331$	0.1674

Table S3. Localization effect of P200. Paired t-test of P200 amplitudes between left (F1, F3, AF3) vs right (F2, F4, AF4) electrode clusters at preexposure and postexposure are performed and corresponding t- value, mean difference (right-left) and $p-$ value are mentioned.

	**Preexposure**		**Postexposure**	
	**Mean difference, t-value**	$p-$**value**	**Mean difference, t-value**	$p-$**value**
**DFE**	$0.1507, t_{11} = 0.1667$	0.4353	$0.4043, t_{11} = 0.6020$	0.2797
**LFE**	$-2.1902, t_{11} = -1.3996$	0.0946	$-1.0275, t_{11} = -0.7005$	0.2491
**NEC**	$1.8796, t_{10} = 3.5183$	0.0028**	$1.1236, t_{10} = 2.2424$	0.0244*

Table S4. Localization effect of N200. Paired t-test of N200 amplitudes between left (P1, P3, PO3) vs right (P2, P4, PO4) electrode clusters at preexposure and postexposure are performed and corresponding t- value, mean difference (right-left) and $p-$ value are mentioned. $^{*}p < 0.05, ^{**}p < 0.01, ^{***}p < 0.001$

	**Preexposure**		**Postexposure**	
	**Mean difference, t-value**	$p-$**value**	**Mean difference, t-value**	$p-$**value**
**DFE**	$-0.0844, t_{11} = -0.1053$	0.4590	$0.4954, t_{11} = 1.0829$	0.1510
**LFE**	$0.4403, t_{11} = 0.5765$	0.2879	$0.6905, t_{11} = 0.8261$	0.2132
**NEC**	$1.1400, t_{10} = 2.4101$	0.0183*	$1.0570, t_{10} = 2.5577$	0.0142*

Table S5. Localization effect of P300. Paired t-test of P300 amplitudes between left (CP1, CP3, P1) vs right (CP2, CP4, P2) electrode clusters at preexposure and postexposure are performed and corresponding t- value, mean difference (right-left) and $p-$ value are mentioned. $^{*}p < 0.05, ^{**}p < 0.01, ^{***}p < 0.001$

	**Preexposure**		**Postexposure**	
	**Mean difference, t-value**	$p-$**value**	**Mean difference, t-value**	$p-$**value**
**DFE**	$-0.0946, t_{11} = -0.1137$	0.4558	$0.0800, t_{11} = 0.1748$	0.4322
**LFE**	$0.4837, t_{11} = 0.5429$	0.2990	$1.2122, t_{11} = 2.0609$	0.0319*
**NEC**	$0.5418, t_{10} = 1.3337$	0.1059	$0.7893, t_{10} = 2.0424$	0.0342*

Table S6. Localization effect of LPP. Paired t-test of LPP between left (CP1, CP3, P1) vs right (CP2, CP4, P2) electrode clusters at preexposure and postexposure are performed and corresponding t- value, mean difference (right-left) and $p-$ value are mentioned. $^{*}p < 0.05, ^{**}p < 0.01, ^{***}p < 0.001$

Table S7 depicted the matrix of partial correlations between ERP amplitudes and participants' desire ratings.

		**Preexposure**					**Postexposure**				
		Desire	P200	N200	P300	LPP	Desire	P200	N200	P300	LPP
DFE	Desire	***1***	**0.30**	**0.30**	-0.18	0.11	***1***	***0.59***	***0.58***	***-0.46***	**0.39**
	P200	**0.30**	***1***	***-0.92*****	***0.44***	-0.21	***0.59***	***1***	***-0.96******	***0.80****	***-0.73****
	N200	**0.30**	***-0.92*****	***1***	***0.56***	-0.18	***0.58***	***-0.96******	***1***	***0.81****	***-0.69****
	P300	-0.18	***0.44***	***0.56***	***1***	***0.68****	***-0.46***	***0.80****	***0.81****	***1***	***0.93******
	LPP	0.11	-0.21	-0.18	***0.68****	***1***	**0.39**	***-0.73****	***-0.69****	***0.93******	***1***
LFE	Desire	***1***	-0.05	-0.27	-0.24	**0.38**	***1***	0.28	0.26	-0.35	***0.43***
	P200	-0.05	***1***	***-0.81****	-0.08	0.13	0.28	***1***	***-0.89*****	0.12	-0.12
	N200	-0.27	***-0.81****	***1***	-0.13	0.23	0.26	***-0.89*****	***1***	0.21	-0.29
	P300	-0.24	-0.08	-0.13	***1***	***0.72****	**-0.35**	0.12	0.21	***1***	***0.73****
	LPP	**0.38**	0.13	0.23	***0.72****	***1***	***0.43***	-0.12	-0.29	***0.73****	***1***
NEC	Desire	***1***	-0.001	-0.25	-0.27	0.06	***1***	0.05	-0.09	-0.08	-0.21
	P200	-0.001	***1***	***-0.89*****	-0.27	0.06	0.05	***1***	***-0.94*****	0.06	-0.23
	N200	-0.25	***-0.89*****	***1***	***-0.42***	0.21	-0.09	***-0.94*****	***1***	-0.02	-0.22
	P300	-0.27	-0.27	***-0.42***	***1***	***0.86*****	-0.08	0.06	-0.02	***1***	***0.72*** *
	LPP	0.06	0.06	0.21	***0.86*****	***1***	-0.21	-0.23	-0.22	***0.72****	***1***

Table S7. Matrix of partial correlations for ERP amplitudes with desire rating at preexposure and postexposure condition. High correlations (i.e, $|r| \geq 0.3$) are indicated in bold. Very high correlations (i.e, $|r| \geq 0.4$) are indicated in bold italic. Multiple testing followed by Benjamini and Hochberg [54] p-value correction was employed.
$^{*}p < 0.05, ^{**}p < 0.01, ^{***}p < 0.001$
